# Characterisation of *HNF1A* variants in paediatric diabetes in Norway using functional and clinical investigations to unmask phenotype and monogenic diabetes

**DOI:** 10.1007/s00125-023-06012-4

**Published:** 2023-10-05

**Authors:** Pernille Svalastoga, Alba Kaci, Janne Molnes, Marie H. Solheim, Bente B. Johansson, Lars Krogvold, Torild Skrivarhaug, Eivind Valen, Stefan Johansson, Anders Molven, Jørn V. Sagen, Eirik Søfteland, Lise Bjørkhaug, Erling Tjora, Ingvild Aukrust, Pål R. Njølstad

**Affiliations:** 1 Mohn Center for Diabetes Precision Medicine, Department of Clinical Science, University of Bergen, Bergen, Norway; 2 Center for Laboratory Medicine, Østfold Hospital Trust, Grålum, Norway; 3 Department of Medical Genetics, Haukeland University Hospital, Bergen, Norway; 4 Division of Childhood and Adolescent Medicine, Oslo University Hospital, Oslo, Norway; 5 Institute of Clinical Medicine, Faculty of Medicine, University of Oslo, Oslo, Norway; 6 Computational Biology Unit, Department of Informatics, University of Bergen, Bergen, Norway; 7 Sars International Centre for Marine Molecular Biology, University of Bergen, Bergen, Norway; 8 Department of Pathology, Haukeland University Hospital, Bergen, Norway; 9 Department of Clinical Medicine, University of Bergen, Bergen, Norway; 10 Department of Medical Biochemistry and Pharmacology, Haukeland University Hospital, Bergen, Norway; 11 Department of Medicine, Haukeland University Hospital, Bergen, Norway; 12 Department of Safety, Chemistry, and Biomedical Laboratory Sciences, Western Norway University of Applied Sciences, Bergen, Norway; 13 Children and Youth Clinic, Haukeland University Hospital, Bergen, Norway

**Keywords:** Diabetes, Functional characterisation, HNF1A-MODY, Maturity onset diabetes of the young, MODY, Monogenic diabetes, Paediatrics, Precision medicine, Sulfonylurea, Variant interpretation

## Abstract

**Aims/hypothesis:**

Correctly diagnosing MODY is important, as individuals with this diagnosis can discontinue insulin injections; however, many people are misdiagnosed. We aimed to develop a robust approach for determining the pathogenicity of variants of uncertain significance in hepatocyte nuclear factor-1 alpha (HNF1A)*-*MODY and to obtain an accurate estimate of the prevalence of HNF1A-MODY in paediatric cases of diabetes.

**Methods:**

We extended our previous screening of the Norwegian Childhood Diabetes Registry by 830 additional samples and comprehensively genotyped *HNF1A* variants in autoantibody-negative participants using next-generation sequencing. Carriers of pathogenic variants were treated by local healthcare providers, and participants with novel likely pathogenic variants and variants of uncertain significance were enrolled in an investigator-initiated, non-randomised, open-label pilot study (ClinicalTrials.gov registration no. NCT04239586). To identify variants associated with HNF1A-MODY, we functionally characterised their pathogenicity and assessed the carriers’ phenotype and treatment response to sulfonylurea.

**Results:**

In total, 615 autoantibody-negative participants among 4712 cases of paediatric diabetes underwent genetic sequencing*,* revealing 19 with *HNF1A* variants. We identified nine carriers with novel variants classified as variants of uncertain significance or likely to be pathogenic, while the remaining ten participants carried five pathogenic variants previously reported. Of the nine carriers with novel variants, six responded favourably to sulfonylurea. Functional investigations revealed their variants to be dysfunctional and demonstrated a correlation with the resulting phenotype, providing evidence for reclassifying these variants as pathogenic.

**Conclusions/interpretation:**

Based on this robust classification, we estimate that the prevalence of HNF1A-MODY is 0.3% in paediatric diabetes. Clinical phenotyping is challenging and functional investigations provide a strong complementary line of evidence. We demonstrate here that combining clinical phenotyping with functional protein studies provides a powerful tool to obtain a precise diagnosis of HNF1A-MODY.

**Graphical Abstract:**

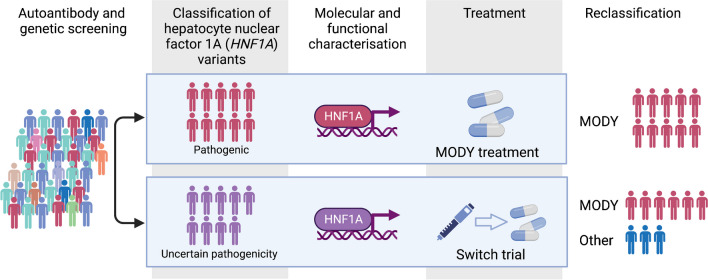

**Supplementary Information:**

The online version contains peer-reviewed but unedited supplementary material available at 10.1007/s00125-023-06012-4.


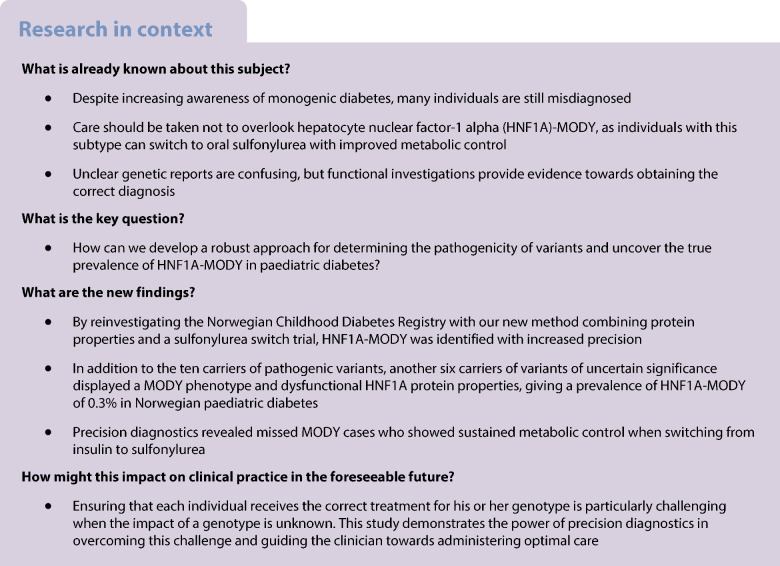


## Introduction

Monogenic diabetes affects up to 6.5% of autoantibody-negative Norwegian children with diabetes aged under 15 years [1, 2], with most cases explained by individuals carrying a single, heterozygous gene variant impairing insulin secretion [3]. The biggest subtype, MODY [4, 5], can be caused by a dysfunction in one of 11 genes, of which the gene encoding hepatocyte nuclear factor-1 alpha (*HNF1A*) serves as the largest contributor in Norway [1]. Hepatocyte nuclear factor-1 alpha (HNF1A)-MODY follows an autosomal dominant inheritance pattern with affected individuals usually found in at least three generations. Individuals typically present with non-ketotic hyperglycaemia and are diagnosed with diabetes in childhood or adolescence [6–9].

MODY is frequently misdiagnosed as cases can be difficult to recognise. This can be explained by overlapping phenotypes with other types of diabetes [1, 10, 11], incomplete penetrance and clinical features that are not always consistent with the classical MODY criteria [12–17]. The autosomal dominant inheritance pattern can also be masked by insufficient clinical information. For this reason, it is essential that systematic screening is performed to uncover misclassified cases that are not picked up on clinical suspicion.

Monogenic diabetes provides a good example where precision diagnostics, including in-depth pathophysiological knowledge, can pave the way for targeted therapy. In diagnosed individuals, sulfonylurea, acting on K_ATP_ channels in pancreatic beta cells, and thereby increasing insulin secretion specifically, improves metabolic control and quality of life [18–22]. Identifying children misclassified as having type 1 diabetes is therefore of great importance, as they can discontinue painful and cumbersome insulin injections, and early detection, when endogenous insulin secretion is highest, translates to a better prognosis when switching to sulfonylurea [23].

The availability of effective treatments has expedited the use of high-throughput sequencing for comprehensive genotyping. While a powerful approach, variant interpretation can still pose a significant challenge. Gene variants are sorted into five categories; pathogenic, likely pathogenic (LP), variant of uncertain significance (VUS), likely benign (LB) or benign [24–27]. Many variants fall into the VUS category due to either limited or conflicting evidence pointing in both benign and pathogenic directions. Furthermore, many disease-causing *HNF1A* variants remain as VUSs due to lack of clinical data and limited or ambiguous results from molecular studies [4]. In the case of a VUS, the individual is left with an unclear molecular diagnosis, and clinicians may misinterpret this result as non-pathogenic. In truth, a classification of VUS simply indicates that more evidence is needed to determine whether or not the variant is pathogenic.

We previously performed a systematic screening of the Norwegian Childhood Diabetes Registry (NCDR), which covers >99% of paediatric diabetes cases in Norway [28], to identify carriers of *HNF1A* variants in autoantibody-negative children [1, 10]. That study gave a lower estimate of prevalence of HNF1A-MODY of 2.2% (excluding VUSs) and an upper estimate of 4.1% (including VUSs). By determining which VUSs are in fact pathogenic, we can not only provide a better treatment for these individuals, but also obtain a more accurate estimate of the true prevalence.

In this study, we extend the scope of our previous search to identify individuals with novel LP variants and VUSs. By combining functional studies of the HNF1A protein with advanced physiological and clinical treatment response data, including a sulfonylurea switch trial, we demonstrate that we can robustly classify these variants. This comprehensive approach can be used to systematically screen and identify HNF1A-MODY and certain other MODY types, and lead to increased diagnostic precision in children with diabetes.

## Methods

We designed an investigator-initiated, non-randomised, open-label pilot study to explore sulfonylurea (ClinicalTrials.gov registration no. NCT04239586) as treatment for HNF1A-MODY in individuals with pathogenic variants, LP variants or VUSs in *HNF1A*. All participants or their legal guardians gave written informed consent, and our research was carried out in accordance with the Declaration of Helsinki. The Western Norway Regional Ethics Committee approved the study (no. 2009/2080 and no. 2018/2388).

### Individuals from genetic screening of the NCDR

NCDR is a Norwegian population-based registry of children aged 0–18 years with newly diagnosed diabetes. By a recent analysis pairing data with the Patient Registry of Norway (http://www.kvalitetsregistre.no, in Norwegian), it was found to cover >99% of paediatric cases and 97.3% of those aged 0–18 years with diabetes in Norway. It is representative for the Norwegian childhood diabetes population regarding sex, ethnicity, age, and regional and socioeconomic factors. At diagnosis, information on ethnicity is collected in addition to clinical and biochemical data, as well as blood samples. From our updated screen we included 830 additional children from the NCDR (diagnosed between March 2015 and July 2017). In addition to the 3882 individuals included in the study by Johansson et al [1], this gave us a total of 4712 participants. Of these, 623 participants had negative autoantibodies (cut-offs: GADA antibody index <0.08 or 1.0 U/ml, IA-2A antibody index <0.1 or 1.0 U/ml) and were subjected to next-generation sequencing (NGS) on an Illumina MiSeq (Illumina, San Diego, CA, USA) sequencer at Hudson Alpha Institute for Biotechnology (Huntsville, AL, USA). The panel set-up included a total of 13 MODY genes (covering all MODY genes recognised to date except *RFX6*): *HNF1A*, *GCK*, *HNF4A*, *HNF1B*, *INS*, *ABCC8*, *KCNJ11*, *BLK*, *CEL*, *NEUROD1*, *KLF11*, *PAX4* and *PDX1*. Detailed information is provided in Johansson et al [1]. As eight participants were excluded from further analysis due to poor DNA quality, the remaining 615 participants (462 [2015] + 153 [2017]) were screened for *HNF1A* variants interpreted according to the ClinGen Monogenic Diabetes Expert Panel specifications to the American College of Medical Genetics and Genomics/the Association for Molecular Pathology (ACMG/AMP) guidelines [27] (electronic supplementary material [ESM] Table 1). Variants classified as benign or LB by the latest guidelines were excluded. For carriers of well-characterised pathogenic variants, we advised local healthcare providers on recommended treatment (sulfonylurea) with a later follow-up by interview in 2021.

Carriers of novel LP variants and VUSs were invited to participate in a sulfonylurea switch trial and physiological assessments in the form of an OGTT. Participants with very low stimulated C-peptide levels on a test meal (C-peptide <0.1 nmol/l) were excluded. One positive control participant, a newly diagnosed carrier of the pathogenic *HNF1A* variant p.Arg203His treated with insulin, was included for comparison in the switch trial.

### Treatment trial of participants carrying novel LP variants and VUSes

#### Re-examination of autoantibody status

At the time of admittance, autoantibodies were measured once again, this time also adding ZnT8A to the two previously tested (cut-offs: GADA: <5 U/ml, IA-2A: <7.5 U/ml, ZnT8A: <15 U/ml).

#### OGTT

Short-acting insulin was discontinued 2 h prior to testing, while long-acting insulin was stopped from 22:00 hours the previous day. Following an overnight fast, 75 g (1.75 g/kg if <40 kg) of glucose (equal to 82.5 g monohydrated) mixed with 200–300 ml of water was ingested at time 0 and plasma was collected at −15, 0, 30, 60, 90 and 120 min for assays of glucose, C-peptide and insulin.

#### Sulfonylurea treatment trial

Glipizide (2.5 mg), a second-generation sulfonylurea, was administered at the 2 h mark of the OGTT. The dose was adjusted gradually according to response over the next few days. Response to sulfonylurea was carefully monitored during the admission. Response was defined as maintaining euglycaemia (finger stick capillary glucose 4.0 to <10.0 mmol/l or 70 to <180 mg/dl) without using insulin within the maximal recommended dosage according to the Norwegian Medicines Agency (https://legemiddelverket.no, accessed multiple times, first time 18 April 2017). Measures were taken to avoid hypoglycaemic incidents during the OGTT and after the switch to sulfonylurea (information, glucose sensors, carbohydrate-rich food at hand, frequent testing of capillary and plasma glucose, and admittance to hospital). In the case of symptomatic hypoglycaemia or random measurement <3.0 mmol/l, testing was to be stopped, and glucose given orally or intravenously. To avoid any risk of ketoacidosis, ketones (capillary and urine) were measured at the 120 min mark of the OGTT, and rechecked until negative in participants with positive measurements. In these participants, reinstatement of insulin was considered early. The study physician (PS) carried out weekly telephone consultations in the first weeks after discharge. Responders were invited for another assessment, with a second OGTT, at a minimum of 3 months after discharge. Participants were instructed to take glipizide before this second assessment.

### Functional studies

#### Construction of *HNF1A* variant plasmids for expression analysis

*HNF1A* variants were constructed using the QuikChange II XL Site-Directed Mutagenesis Kit (Agilent Technologies, Santa Clara, CA, USA) and variant-specific primers. Individual *HNF1A* variants were introduced into the wild-type *HNF1A* cDNA isoform A (NCBI NM_000545.6, with substitutions; c.51C>G, p.Leu17= and c.79A>C, p.Ile27Leu) in the pcDNA 3.1/HisC vector. In the transactivation assays, the firefly luciferase reporter vector pGL3-RA (Promega, Madison, WI, USA), containing the rat albumin promoter cloned into a pGL3 vector, as well as the *Renilla* luciferase control vector pRLSV40 (Promega) were used as described previously [24, 26].

#### Luciferase reporter assays and protein abundance

HeLa cells were cultured and transiently transfected with wild-type or variant *HNF1A* cDNA, together with the firefly reporter plasmid and the *Renilla* reporter (pRLSV40) as an internal control. Luciferase activity was measured 24 h post transfection using the Dual-Luciferase Assay System (Promega) on a Centro XS3 LB 960 luminometer (Berthold Technologies, Germany). Next, the level of HNF1A protein expression in the wild-type and variants was assessed in cell lysates obtained for the transactivation assays. In short, 20 µl of cell lysates was subjected to SDS-PAGE and immunoblotting using antibodies against HNF1A (Cell Signaling, Beverly, MA, USA) and α-tubulin (Abcam, Cambridge, MA, USA), with α-tubulin as a loading control. For the five pathogenic variants, these variants have been comprehensively investigated previously [25, 26, 29, 30] and thus only the luciferase assay was reinvestigated.

#### Nuclear fractionation

Nuclear and cytosolic fractions were isolated from transiently transfected HeLa cells as previously performed [31]. Total protein in each fraction was measured using Bradford reagent (Thermo Fisher, Waltham, MA, USA) and 8 µg of total protein from each fraction was subjected to SDS-PAGE and immunoblotting with antibodies for HNF1A (Cell Signaling). The relative subcellular localisation was calculated by using the ratios of HNF1A with the respective nuclear (topoisomerase IIα [Abcam]) and cytosolic (α-tubulin [Abcam]) markers.

#### DNA binding studies

The electrophoretic mobility shift assays were carried out as previously described [31]. Briefly, equal protein amounts of nuclear fractions from transiently transfected HeLa cells were incubated together with a cyanine 5-labelled oligonucleotide (Sigma Aldrich, St Louis, MO, USA), using the Odyssey EMSA buffer kit (LI-COR Biosciences, Lincoln, NE, USA) and the promoter of the rat *Alb* gene (5′-TGTGGTTAATGATCTACAGTTA-3′) for the binding reaction.

### Statistical analysis

We present results as means ± SD and relative to wild-type HNF1A (set to 100%), unless stated otherwise. Statistical differences were analysed using two-tailed Student’s *t* tests with a significance level of *p*<0.05, using GraphPad Prism software version 8.1.1 (GraphPad Software, San Diego, CA, USA).

## Results

### Clinical assessments

**Fig. 1:**
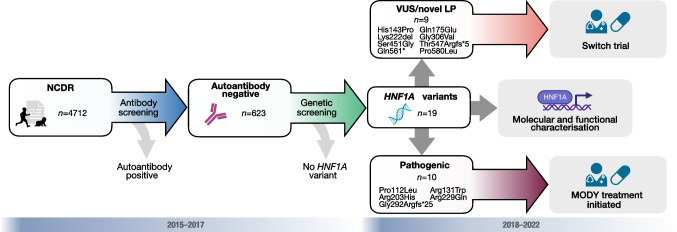
Timeline for screening *HNF1A* variants in the NCDR. The NCDR was screened in 2015 and 2017, including a total of 4712 children with diabetes. Of these, 623 participants were autoantibody negative and eligible for genetic analysis, but only 615 of these samples displayed high-quality DNA or coverage. Ten participants with five different pathogenic variants were identified, and these were switched to MODY treatment (sulfonylurea) by local paediatricians. An additional nine carriers of novel LP variants or VUSs were invited to an inpatient switch trial in which insulin was replaced with sulfonylurea, with investigation of the phenotype and response to treatment. Molecular and functional investigations of participants’ *HNF1A* variants complemented the clinical assessments

Of the 4712 children with diabetes from the NCDR, 615 were GADA and IA-2A negative and subjected to genetic screening. Of these, 19 participants were identified as carrying *HNF1A* variants classified as pathogenic, LP or VUS (Fig. 1). Nine carriers of eight different novel LP variants or VUSs in *HNF1A* were identified (Table 1) and included in the switch trial. Their diagnoses had not been confirmed but two participants were treated with sulfonylurea on clinical suspicion of MODY. The remaining ten participants carried variants already established as pathogenic and were, as expected, sensitive to diet or insulin secretagogues (ESM Table 2). Only one carrier in this group was not previously diagnosed with HNF1A-MODY.

**Table 1:** Characteristics of children carrying *HNF1A* novel LP variants and VUSs

Variable	Successful switch						Unsuccessful switch		
Nucleotide change	c.428A>C	c.666_668del	c.666_668del	c.1640_1641del	c.1351A>G	c.1681C>T	c.523C>G	c.917G>T	c.1739C>T
Amino acid change	p.His143Pro	p.Lys222del	p.Lys222del	p.Thr547Argfs*5	p.Ser451Gly	p.Gln561*	p.Gln175Glu	p.Gly306Val	p.Pro580Leu
Family history	No	Yes	Yes	No	Yes	NA	Yes	No	No
Co-segregation	Yes	Yes	Yes	Inconclusive	Inconclusive	Inconclusive	No	No	No
Sex	Male	Female	Male	Male	Female	Female	Male	Male	Male
Age at diagnosis, years	14	11	13	11	13	11	11	6	7
Age at assessment, years	20	29	23	16	24	14	20	13	20
GADA/IA-2A/ZnT8A	Neg/Neg/Neg	Neg/Neg/Neg	Neg/Neg/Neg	Neg/Neg/Neg	Neg/Neg/Neg	Neg/Neg/Neg	Neg/Neg/Neg	Pos/Neg/Neg	Neg/Neg/Neg
Insulin, U/kg per day	0.4	0.6	NA	0.2	NA	0.4	1.5	0.9	1.1
BMI, kg/m^2^	28.1	29.7	29.0	27.0	23.8	26.6	29.7	19.9	19.3
Fasting C-peptide, nmol/l	0.30	0.38	0.54	0.53	1.02	0.66	0.36	<0.03	0.01
Stimulated^a^ C-peptide, nmol/l	0.89	0.76	2.02	1.45	1.70	3.13	0.87	0.12	0.01
HOMA-IR	4.6	5.6	1.7	3.5	9.5	5.0	19.9	1.3	1.8
HbA_1c_^b^, mmol/mol	78	68	NA	52	NA	51	65	64	75
HbA_1c_^b^, %	9.3	8.4	NA	6.9	NA	6.8	8.1	8.0	9.0
HbA_1c_^c^, mmol/mol	73	NA	65	42	54	48	NA	NA	NA
HbA_1c_^c^, %	8.9	NA	8.1	6.0	7.1	6.5	NA	NA	NA
Glipizide, mg/d	24.0	NA	7.0^d^	5.2^d^	7.5^d^	1.7	NA	NA	NA
Phenotype interpretation	MODY	MODY	MODY	MODY	MODY	MODY	Type 2	Type 1	Type 1

Reference transcript (*HNF1A*): NM_000545.6 (https://www.ncbi.nlm.nih.gov/nuccore/807201168)

^a^Stimulated, by glucose during OGTT

^b^Before the switch trial

^c^After the switch trial, at least 3 months

^d^Equivalent glipizide dose, calculated using the conversion table from the study by Farahani [43]

NA, not applicable; Neg, negative; Pos, positive

**Fig. 2:**
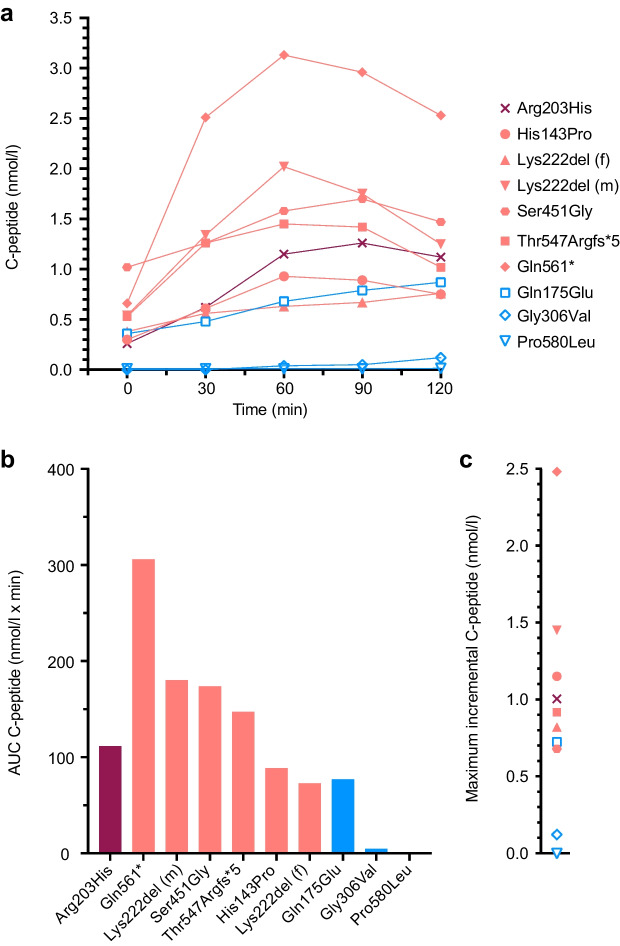
C-peptide measurements from the OGTT. (**a**) C-peptide levels in the variant carriers during a 2 h OGTT. Participants included in the switch trial showed heterogeneous insulin secretion. Carriers are coloured according to the response to sulfonylurea: switched (light red) or not switched (blue) vs the pathogenic control participant (dark red). The carrier of p.Pro580Leu was excluded from the switch trial and the results depicted are a stimulated C-peptide test. (**b**) AUC for participants during the OGTT, sorted by decreasing numbers within the groups. (**c**) Maximum incremental C-peptide for participants. The p.Lys222del variant carriers are siblings, and the individual with a lower insulin response (up-pointing triangles) is the female sibling detailed in Table 1

In the treatment switch trial, six of the nine participants responded to sulfonylurea and three responded poorly to the switch, judged by a failed stimulated C-peptide test pre-admission or an insufficient response to sulfonylurea. Of the six participants with preserved insulin secretion (Fig. 2a–c), careful examination revealed co-segregation (variant and phenotype appear in the same individuals in a family) of the variant and diabetes in only three families, the rest being inconclusive due to missing clinical/family information (p.Gln561*), multiple diabetes phenotypes in the family (p.Ser451Gly) and reduced penetrance in the parent carrier (p.Thr547Argfs*5) (ESM Fig. 1). Reassessing the participants who successfully switched revealed sustained or improved HbA_1c_ in these individuals at follow-up (ESM Fig. 2), and a participant with a continuous glucose monitor also showed improvement in time in range [32]) (ESM Fig. 3). One individual who successfully switched was lost to follow-up.

Of the three non-responsive VUS carriers, the p.Gln175Glu carrier was of South Asian heritage and overweight with an elevated insulin requirement before switch and raised HOMA-IR, indicating insulin resistance and type 2 diabetes (Table 1). In the switch trial, sulfonylurea failed as monotherapy at the maximum recommended dose and insulin was started at day 2. Oral sulfonylurea was withdrawn after only 2 weeks. The two other participants failing the switch attempt (carriers of p.Gly306Val and p.Pro580Leu) revealed almost no insulin secretion, both showing a type 1 diabetes phenotype, and one developed elevated GADA during the study period. Lineage studies revealed a lack of co-segregation, contradicting HNF1A-MODY, in these participants (ESM Fig. 1).

### Functional studies

**Fig. 3:**
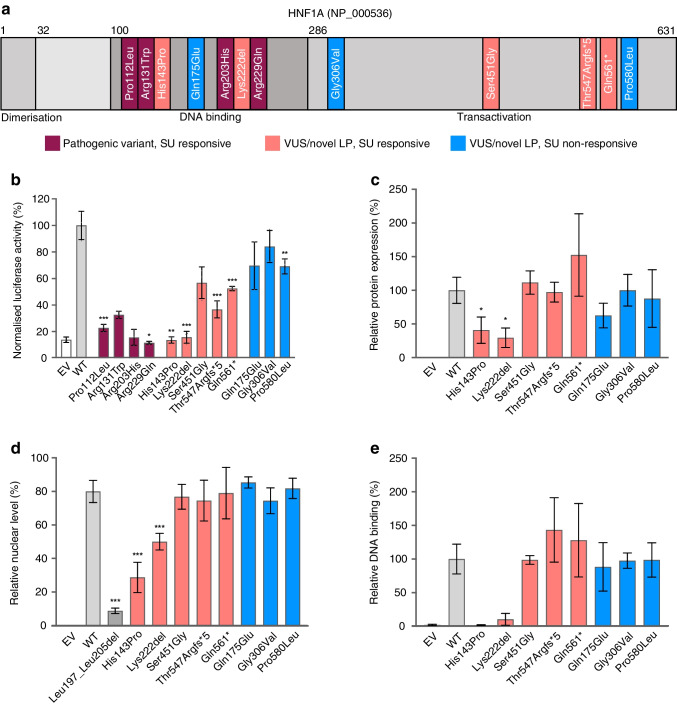
Functional investigation of *HNF1A* VUSs, LP and pathogenic variants identified through screening of antibody-negative individuals in the NCDR. (**a**) Schematic illustration of the HNF1A protein with the positions of the identified variants. The dimerisation, DNA binding and transactivation domains of the HNF1A protein are highlighted. The pathogenic variant p.Gly292Argfs*25 is not included for reasons described in the main text. (**b**) Assessment of transcriptional activity of HNF1A protein variants using a luciferase reporter assay. HeLa cells were transiently transfected with wild-type or variant *HNF1A* plasmids together with the reporter plasmids encoding firefly (pGL3-RA) and *Renilla* (pRLSV40) luciferase. (**c**) Relative protein expression. HeLa cell lysates collected for the transactivation assay were analysed by SDS-PAGE and immunoblotting using HNF1A-specific antibodies. Protein levels, normalised to α-tubulin (loading control), are presented relative to wild-type levels (set as 100%). Representative western blots are shown in ESM Fig. 4a. (**d**) Nuclear localisation of HNF1A variants. Nuclear fractions of transiently transfected HeLa cells (wild-type or variant *HNF1A* plasmids) were assessed by SDS-PAGE immunoblotting. p.Leu197_Leu205del was used as a negative control [44], and topoisomerase IIα and α-tubulin were used as nuclear and cytosolic markers, respectively. The HNF1A/topoisomerase IIα and HNF1A/α-tubulin ratios were used to calculate the relative subcellular localisation of HNF1A in each compartment. Representative western blots are shown in ESM Fig. 4b. (**e**) DNA binding of HNF1A variants in an electrophoretic mobility shift assay. Equal amounts of *HNF1A* variants in nuclear fractions were incubated with a cyanine 5-labelled oligonucleotide corresponding to the HNF1A binding site and bound complexes were quantified by densiometric analysis (representative gel images are shown in ESM Fig. 4c). Measurements are given relative to wild-type levels (set as 100%) unless otherwise specified. Each bar represents the mean of nine readings ±SD; three parallel readings were conducted on three experimental days. *p<0.05, **p<0.01, ***p<0.001. Dark red bars: four pathogenic variants in participants identified in the screening study. Light red bars: novel LP variants or VUSs in successfully switched participants. Blue bars: VUSs in non-switched participants. EV, empty vector; SU, sulfonylurea; WT, wild-type

The positions of the 12 *HNF1A* variants (four pathogenic, two novel LP variants and six VUSs) in the HNF1A protein domain, and their effect on normal HNF1A transcriptional activity, protein abundance, nuclear localisation and DNA binding ability, are shown in Fig. 3a–e (corresponding western blots in ESM Fig. 4). The pathogenic variant p.Gly292Argfs*25 was not included in the functional investigation as it was previously shown to induce a frame shift, with a premature stop codon triggering nonsense-mediated decay of the RNA and preventing the truncated protein from being made [33]. Of the 12 remaining variants, nine (p.Pro112Leu, p.Arg131Trp, p.His143Pro, p.Arg203His, p.Lys222del, p.Arg229Gln, p.Ser451Gly, p.Thr547Argfs*5 and p.Gln561*) demonstrated reduced levels of transcriptional activity (12–57% of wild-type), while three variants (p.Gln175Glu, p.Gly306Val and p.Pro580Leu) demonstrated only mildly reduced activity (70–85% of wild-type). We observed significantly lower protein expression levels for the p.His143Pro and p.Lys222del variants (41% and 30%, respectively) compared with wild-type expression levels. These two variants also exhibited a significant reduction in nuclear protein level and severely reduced DNA binding (<10%). Co-expression of increasing quantities of p.Thr547Argfs*5 or p.Gln561* with fixed levels of wild-type HNF1A excluded a dominant negative effect for these variants (ESM Fig. 5).

## Discussion

In the present study, we show that 16 of 623 (2.6%) Norwegian children with antibody-negative diabetes (representing 0.3% of the NCDR) carry variants leading to impaired protein function of HNF1A, with phenotypic and functional investigations supporting HNF1A-MODY. In six cases, genetic screening revealed a novel LP variant or a VUS where further characterisation showed strong indications of HNF1A-MODY, with sustained metabolic control after switching to sulfonylurea. Our studies demonstrate that having an unclear genetic report entails a risk of being misdiagnosed and ineffectively treated with insulin. Screening for HNF1A-MODY, in addition to variant characterisation and a sulfonylurea trial, can correct a misdiagnosis.

Clinical phenotyping is challenging and an atypical presentation at the time of referral has contributed to monogenic diabetes being unrecognised, with people being clinically diagnosed as having type 1 diabetes and treated with insulin. In many cases, there was no family history (e.g. missing data, age-dependent penetrance, de novo mutation) to indicate autosomal dominant inheritance. Together, this highlights the complexity of clinical diagnostics and demonstrates the need for investigation by systematic screening and characterisation of carriers of novel variants or VUSs.

In agreement with previous reports on functional analyses of other variants [7, 8, 24, 25, 30], loss of transactivation in p.Thr547Argfs*5 and p.Gln561* variants cannot be explained by impaired DNA binding ability, nuclear targeting or reduced protein expression as these subanalyses are not affected. In contrast, impaired protein function in p.His143Pro and p.Lys222del might be explained by the combined effect of several mechanisms. In this study, the p.Ser451Gly variant demonstrated only moderately reduced transcriptional activity (~60%) and did not impede any other HNF1A function. Although the clinical presentation correlated with a MODY phenotype in this individual, it is unclear whether the variant alone is causing a highly penetrant diabetes, or whether the effect could be driven in a polygenic manner, as the segregation studies were inconclusive, with a high proportion of type 2 diabetes cases in non-carriers in the family.

Consistent with sulfonylurea insensitivity in p.Gln175Glu, p.Gly360Val and p.Pro580Leu, the transactivation assays demonstrated only minor reduction (70–85% of wild-type) and no indication of pathogenic variants, indicating the presence of other diabetes subtypes in the respective carriers. This illustrates an important message that pathogenicity cannot be determined for VUS carriers, and careful characterisation of the variant is needed. Removing insulin from people with atypical type 1 diabetes (negative autoantibodies) comes with a great risk of ketoacidosis, so careful intervention in a hospital setting is critical when assessing variant carriers and treatment response in paediatric patients. In these non-responding individuals, the transactivation assay of these specific variants alone pointed to other causes of diabetes.

A strength of our study is the completeness of the NCDR, making our population-based findings representative of a northern European population, like a similar recent registry study from the Nordic region [34]. Unlike studies estimating prevalence by genetically screening only referred individuals or small subsets of individuals, our study is less affected by selection bias [11, 35–39]. The use of a larger NGS gene panel, containing 16 genes, in addition to selected promoter regions and the inclusion of VUSs makes it unlikely that any individual was missed in our screen. Another advantage is the robust variant interpretation guidelines used in this study [27].

Because of the rarity of the disorder, the number of individuals carrying *HNF1A* variants is small, even in a nationwide cohort. At the time of inclusion, the NCDR only contained children under the age of 15 years, and our lower prevalence compared with similar screening studies (0.5–1.0%) is likely a reflection of our younger cohort [40, 41]. This could be addressed in future screening trials, as the age limit of the registry has now been increased to 18 years. Ideally, all variant carriers would follow the same protocol, but as the switch study was initiated years after the screening study, many carriers had already been receiving sulfonylurea. We did, however, manage to prospectively enrol one insulin-treated carrier with a pathogenic variant who was successfully switched to sulfonylurea. In our study, we simply illustrate the large variation in insulin secretion (Fig. 2). We realise that the heterogeneity and progressiveness of HNF1A-MODY makes any comparison complex. Phenotypes of diabetes tend to overlap, and many factors affect insulin secretion, importantly age and diabetes duration, as previous studies have already established [42].

As we included only autoantibody-negative individuals in the screening study, although a reasonable selection, we risked missing a few individuals, as the specificity of antibody assays is never 100% and some individuals with MODY might be autoantibody-positive with or without related autoimmune diabetes. A potential compromise could be to include individuals with low titre autoantibodies, such as in the study by Harsunen et al [34]. We did, however, investigate the autoantibody-positive VUS carriers from Johansson et al [1] (ESM Table 1), finding little indication of HNF1A-MODY in the clinical evaluation of the specific variants (p.Ser22Arg and p.Thr354Met). In further support of p.Thr354Met being benign, our previous functional investigation of the p.Thr354Met variant [25] also did not support a damaging effect on protein function. The ACMG and the AMP evidence for these two variants is provided in ESM Table 1, and functional analyses are provided in a previous publication [25] and ESM Fig. 6. The p.Ser22Arg variant was detected in two autoantibody-positive individuals, but only one gave consent to participate in the study. The consenting participant was triple autoantobody-positive (GADA/IA-2A/ZnT8 11.4/241/333 U/ml [cut-offs: GADA: <5 U/ml, IA-2A: <7.5 U/ml, ZnT8A: <15 U/ml]), revealing no glucose-stimulated endogenous insulin secretion in an OGTT (2 h serum C-peptide <0.03 nmol/l, 2 h serum glucose 23 mmol/l). The father, aged 53 years, also carries the variant but does not have diabetes. There is no other history of diabetes in the family. However, as we found a discrepancy in the clinical and functional assessments of the p.Ser22Arg variant, further investigations, including a specific dimerisation assay, would be of interest. Also, follow-up of the father regarding diabetes status in the years to come might reveal pathogenicity with a lower penetrance, and if the proband has type 1 diabetes exclusively or double diabetes (HNF1A-MODY and type 1 diabetes). Because of these uncertainties, the variant remains a VUS after this study. However, it is notable that the allele frequency of this variant in the Genome Aggregation Database (gnomAD) is higher than one would expect in a MODY-causing variant (minor allele frequency 0.013%, 37 allele counts).

Identifying and correctly diagnosing HNF1A-MODY clinically is difficult due to the challenge of phenocopies, and sulfonylurea response alone is not a suitable diagnostic marker. Our thorough approach addresses this, thus limiting the risk of misdiagnosis for carriers of *HNF1A* variants. First, this approach improves discovery by screening for HNF1A-MODY in high-risk populations and, second, it combines clinical and functional investigations into a robust classification scheme that increases precision in diagnosing monogenic diabetes. Specifically, we have demonstrated that functional studies aid in determining the pathogenicity of novel variants and VUSs.

In summary, we present a comprehensive method to classify paediatric cases of HNF1A-MODY, which was used to determine the pathogenicity of novel variants and VUSs. Our approach is readily extendable to variants occurring in similar diabetes-implicated transcription factors. The impact of obtaining a correct diagnosis in these individuals is considerable, permitting insulin-free treatment and the possibility of testing at-risk family members. This brings about an important change in management in these children, improving quality of life and reducing the risk of diabetes-associated late complications.

### Supplementary Information

Below is the link to the electronic supplementary material.

Supplementary file1 (PDF 4869 KB)

## Data Availability

The datasets analysed during the current study are available from the corresponding author on reasonable request.

## Abbreviations

ACMG: American College of Medical Genetics and Genomics
AMP: Association for Molecular Pathology
HNF1A: Hepatocyte nuclear factor-1 alpha
LB: Likely benign
LP: Likely pathogenic
NCDR: Norwegian Childhood Diabetes Registry
NGS: Next-generation sequencing
VUS: Variant of uncertain significance
