# Supplementary material for: Characterisation of HNF1A variants in paediatric diabetes in Norway using functional and clinical investigations to unmask phenotype and monogenic diabetes
Source: Diabetologia. 2023 Oct 5;66(12):2226–37. doi: 10.1007/s00125-023-06012-4 (PMC10627920; doi:10.1007/s00125-023-06012-4)
Supplement: Supplementary file 1 — Supplementary file1 (PDF 4869 KB) [file 125_2023_6012_MOESM1_ESM.pdf]

# Electronic Supplementary Material (ESM)

**Characterisation of *HNF1A* variants in paediatric diabetes in Norway using functional and clinical investigations to unmask phenotype and monogenic diabetes.**

## *ESM Tables*


Page 2 - ESM Table 1 - Summary of the ACMG scoring

Page 3 - ESM Table 2 - Overview of variants and treatment


## *ESM Figures*


Page 4 - ESM Fig. 1 - Carrier status of subjects

Page 5 - ESM Fig. 2 - $HbA_{1c}$ Before and after switch

Page 6 - ESM Fig. 3 - Diurnal metabolic control on sulfonylurea

Page 7 – ESM Fig. 4 - Representative western blots for the functional studies

Page 8 - ESM Fig. 5 - Dominant negative effect on HNF1A transcriptional activity

Page 9 - ESM Fig. 6 - Functional studies on p.Ser22Arg in autoantibody-positive carriers

Page 10 - References

# ESM TABLES

| Nucleotide change | Amino acid change | ACMG-AMP evidence | Classification |
|---|---|---|---|
| c.66C>G | p.Ser22Arg | PM1_supporting, PP3 | VUS |
| c.335C>T | p.Pro112Leu | PS4, PS3_moderate, PM1_supporting, PM2_supporting, PP1_strong, PP3, PP4_moderate | Pathogenic† |
| c.391C>T | p.Arg131Trp | PS4, PM2_supporting, PM1, PM5, PP3, PP4_moderate, PP1_strong | Pathogenic† |
| c.428A>C | p.His143Pro | PM1, PM2_supporting, PM5_supporting, PP3 | VUS |
| c.523C>G | p.Gln175Glu | PM2_supporting | VUS |
| c.608G>A | p.Arg203His | PS4, PS3_supporting, PM1, PM2_supporting, PM5_supporting, PP3, PP4_moderate | Pathogenic† |
| c.666_668del | p.Lys222del | PM1_supporting, PM2_supporting, PM4_supporting, PP4_moderate | VUS† |
| c.686G>A | p.Arg229Gln | PS4, PS3_supporting, PM1_supporting, PM2_supporting, PM5_supporting, PP3, PP1_strong, PP4_moderate | Pathogenic† |
| c.872dup | p.Gly292Argfs*25 | PVS1, PP1_strong, PS2_moderate, PP4_moderate | Pathogenic† |
| c.917G>T | p.Gly306Val | PM2_supporting | VUS |
| c.1061C>T | p.Thr354Met | BS1_strong | VUS |
| c.1351A>G | p.Ser451Gly | PM2_supporting, PP3 | VUS |
| c.1640_1641del | p.Thr547Argfs*5 | PVS1, PM2_supporting | Likely pathogenic |
| c.1681C>T | p.Gln561* | PVS1, PM2_supporting | Likely pathogenic |
| c.1739C>T | p.Pro580Leu | PM2_supporting | VUS |

**ESM Table 1. Summary of the ACMG scoring performed on the *HNF1A* variants prior to functional investigations and switch trial.** *HNF1A* variants (NM_000545.6) with description of predicted changes at protein level with interpretations according to the ClinGen Monogenic Diabetes Variant Curation Expert Panel (MDEP) *HNF1A* gene specifications to the American College of Medical Genetics and Genomics and the Association for Molecular Pathology (ACMG-AMP) guidelines. †Also recently reviewed and deposited in ClinVar as pathogenic or likely pathogenic (Lys222del) by the MDEP (VCI approved). VUS: Variant of Uncertain Significance.

| Nucleotide change | Amino acid change | Classification | *n* | Treatment before the switch study |
|---|---|---|---|---|
| c.335C>T | p.Pro112Leu | Pathogenic | 3 | Sitagliptine<br>Diet<br>SU |
| c.391C>T | p.Arg131Trp | Pathogenic | 1 | SU and Insulin |
| c.428A>C | p.His143Pro | VUS | 1 | Insulin |
| c.523C>G | p.Gln175Glu | VUS | 1 | Insulin |
| c.608G>A | p.Arg203His | Pathogenic | 2 | SU<br>SU |
| c.666_668del | p.Lys222del | VUS | 2 | Insulin<br>SU |
| c.686G>A | p.Arg229Gln | Pathogenic | 2 | SU and Insulin<br>SU |
| c.872dup | p.Gly292Argfs*25 | Pathogenic | 2 | Diet<br>Diet |
| c.917G>T | p.Gly306Val | VUS | 1 | Insulin |
| c.1351A>G | p.Ser451Gly | VUS | 1 | SU |
| c.1640_1641del | p.Thr547Argfs*5 | Likely Pathogenic | 1 | Insulin |
| c.1681C>T | p.Gln561* | Likely Pathogenic | 1 | Insulin |
| c.1739C>T | p.Pro580Leu | VUS | 1 | Insulin |

**ESM Table 2. Overview of variants and treatment.** Variants in *HNF1A* classified as variants of uncertain significance (VUS), likely pathogenic- or pathogenic identified through screening of antibody negative individuals in the Norwegian Childhood Diabetes Registry. Reference transcript (*HNF1A*): NM_000545.6. SU: sulfonylurea.

# ESM FIGURES

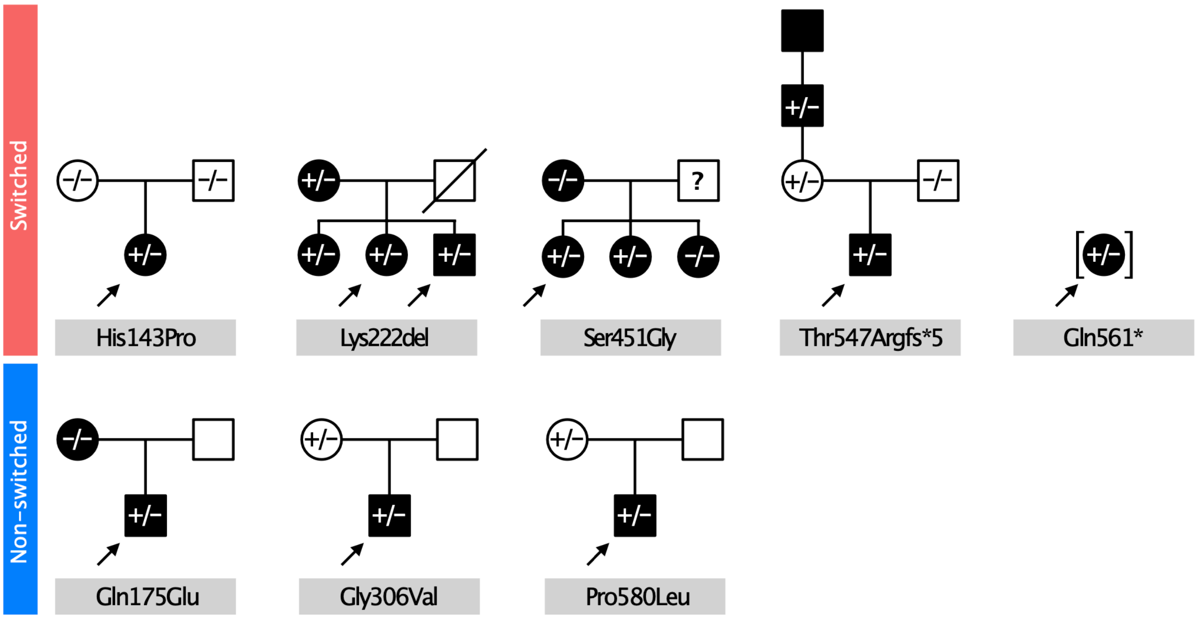


**ESM Fig. 1. Family pedigrees of individuals with novel likely pathogenic variants and variants of uncertain significance.** Switched subjects (upper, from the left): His143Pro: The index had a *de novo* mutation. Lys222del: The variant co-segregated with diabetes, with two carriers included in the study. Ser451Gly: Co-segregation data is inconclusive because of missing clinical information and multiple diabetes phenotypes in the family. Thr547Argfs*5: Low penetrance in parent-carrier (age 41 yrs.) makes co-segregation data inconclusive. Gln561*: The index was adopted, so co-segregation is inconclusive.

Non-switched subjects (lower, from the left): No co-segregation was found in these families.

Arrows indicate individuals identified through the screening, included in the switch study. Black colour indicates diabetes. +/- : Heterozygosity of the variant. -/-: Non-carrier. ? indicates unknown phenotype (diabetes status).

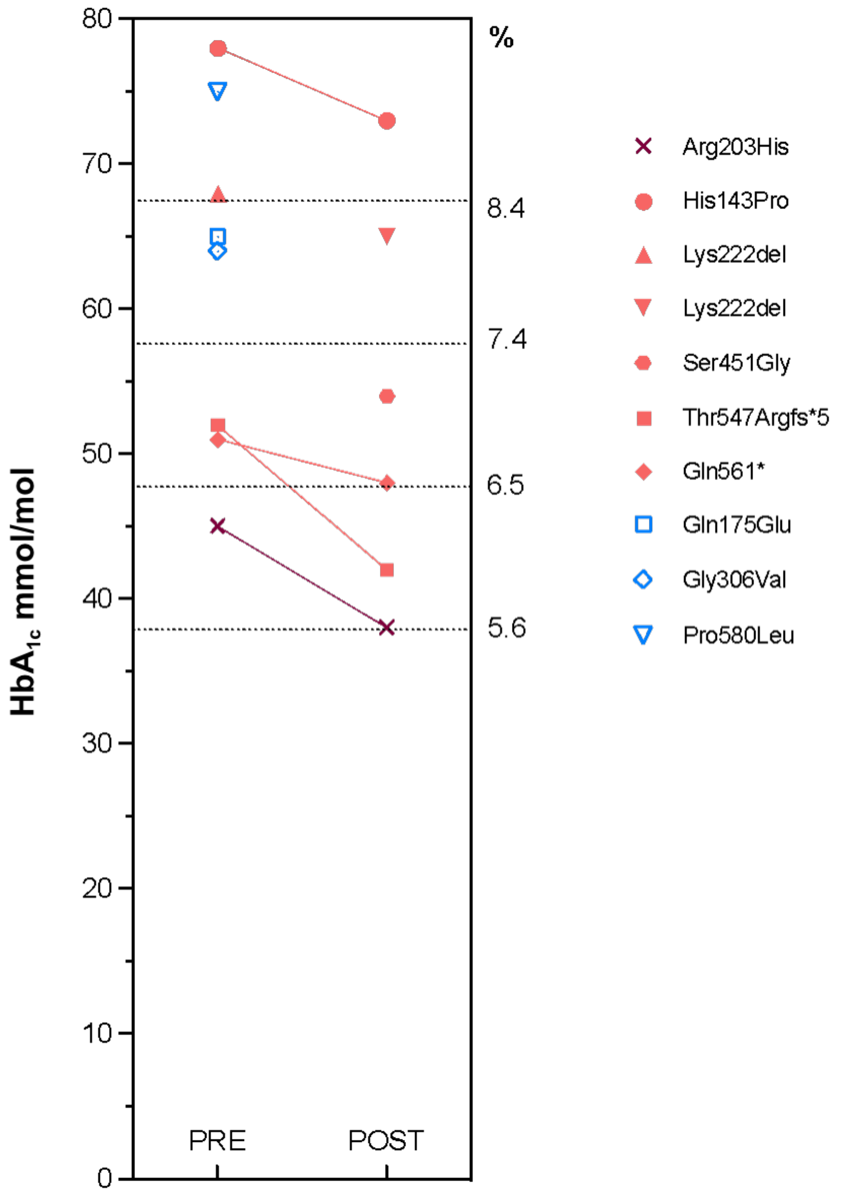


**ESM Fig. 2. Glycated haemoglobin levels associated with switching from insulin to sulfonylurea therapy.** $HbA_{1c}$ levels (mmol/mol) in the carriers, recorded at the first assessment (PRE), and reassessment (POST) after switching to sulfonylurea. The second OGTT was performed 12 - 27 months after the first OGTT in the three individuals reassessed. Two individuals (non-index p.Lys222del and p.Ser451Gly) were already using sulfonylurea. Carriers only included in the "PRE" column proved non-responsive to sulfonylurea (blue) or dropped out (index p.Lys222del). Dark red: Pathogenic variant (positive control). Light red: carriers of novel LP/VUS responsive to sulfonylurea. Blue: VUS-carriers unresponsive to sulfonylurea. Right y-axis illustrates $HbA_{1c}$ measures in %.

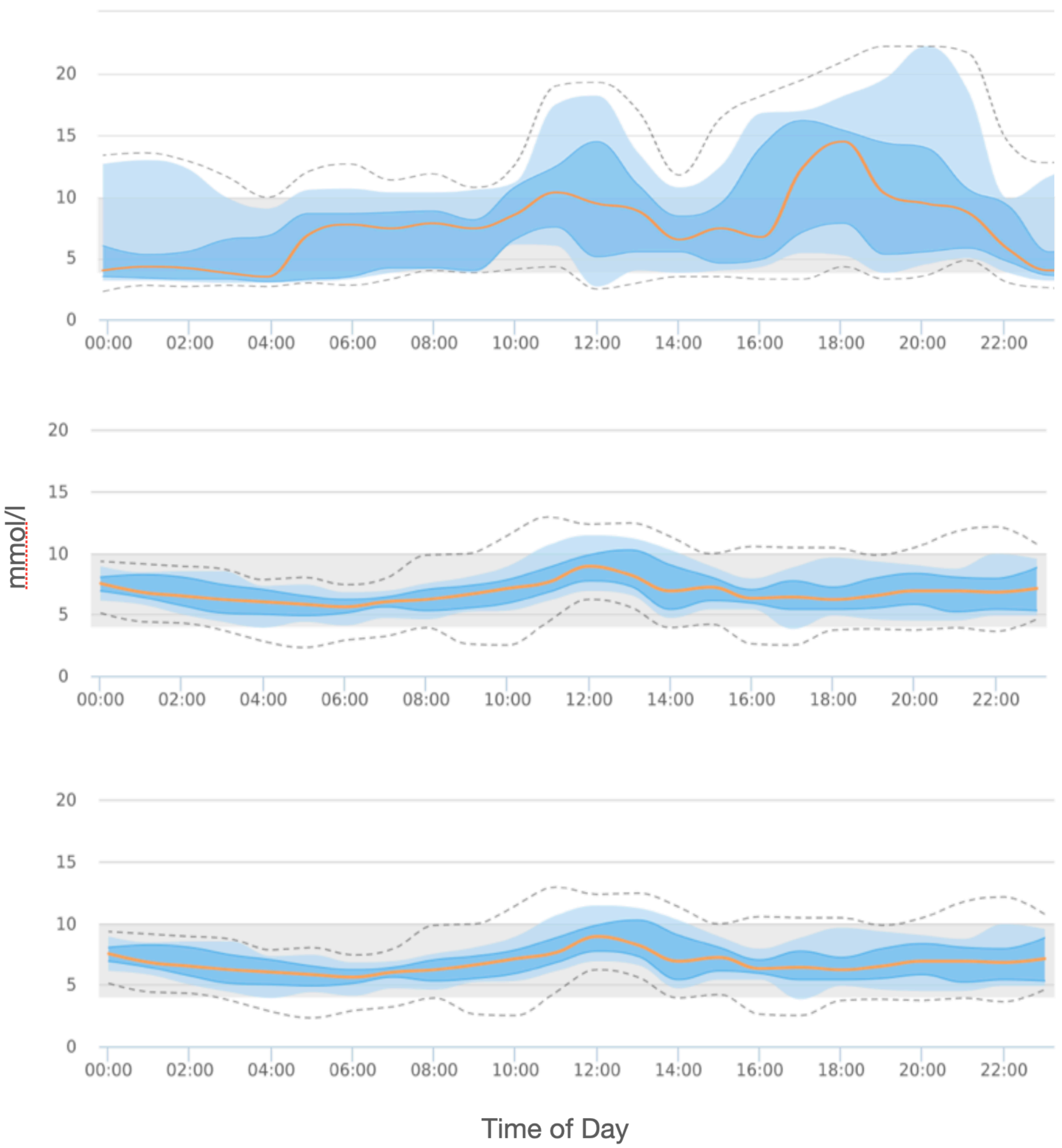


**Fig. 3. Diurnal metabolic control on sulfonylurea.** Shown are data from 14 days using a continuous glucose monitoring system (Dexcom G4™). Upper panel reveals an individual with type 1 diabetes ($HbA_{1c}$ 50 mmol/mol) included for comparison, while the middle panel and the lower panel are from two different 14-day periods of the individual carrying the variant p.Thr547Argfs*5 ($HbA_{1c}$ 52 mmol/mol) during the first month after the switch from insulin to sulfonylureas. The data are displayed as mean (orange line), interquartile range (IQR, 25-75%, blue), range from 10th to 90th centile (10-90%, light blue) of all monitored values during day and night for 14 days. According to the consensus recommendations from the Advanced Technologies & Treatments for Diabetes (ATTD) Congress, target ranges for tissue glucose measured by continuous glucose monitoring is more than 70% of the values to be in the 4 - 10 mmol/l range (displayed in grey) (1).

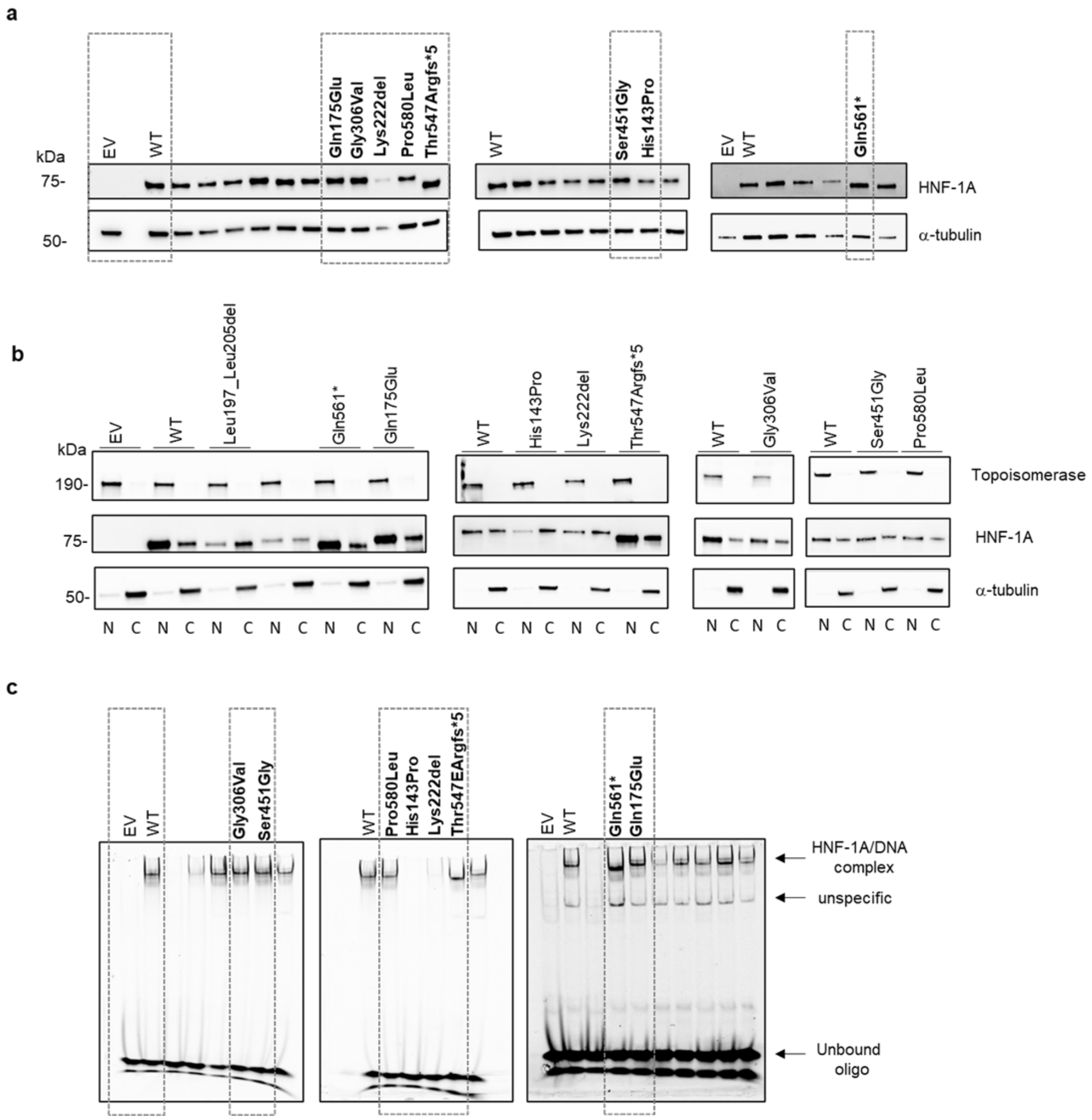


**ESM Fig. 4. Western Blots and EMSA gels for the functional studies of HNF1A protein variants.** **(a)** shows HNF1A protein levels, **(b)** nuclear localisation of HNF1A protein variants, and (**c**) shows electrophoretic mobility shift assays of HNF1A protein variants.

a

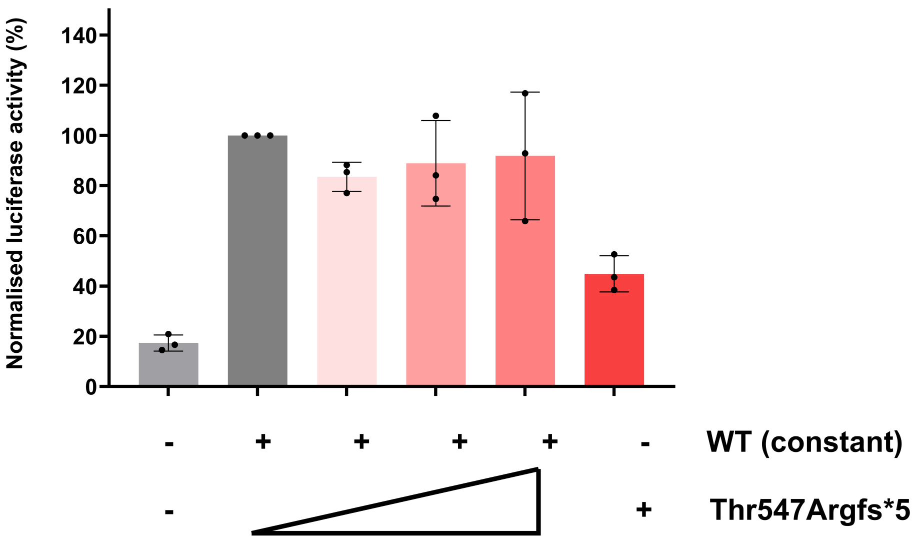


b

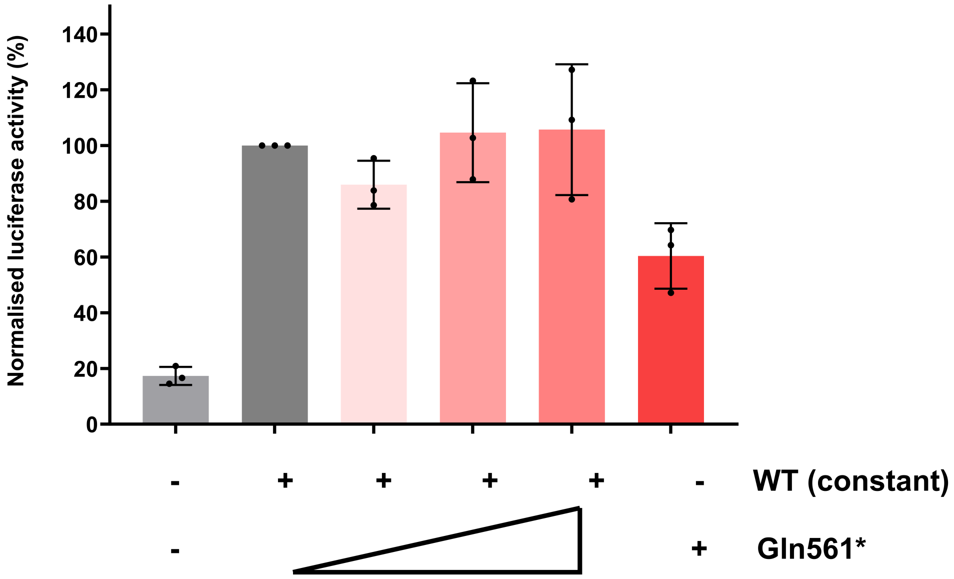


**ESM Fig. 5. The p.Thr547Argfs*5 and p.Gln561* variants did not exert a dominant negative effect on wild-type HNF1A transcriptional activity.** HeLa cells were transiently co-transfected with a constant amount of wild-type and increasing amounts of p.Thr547Argfs*5 (**a**) or p.Gln561* (**b**) together with the reporter plasmids encoding *Firefly* (pGL3-RA) and *Renilla* (pRL-SV40) luciferase. Measurements are given relative to wild-type activity (set as 100%). Each bar represents the mean of nine readings ± standard deviation; three parallel readings conducted on each of three experimental days (*n*=3).

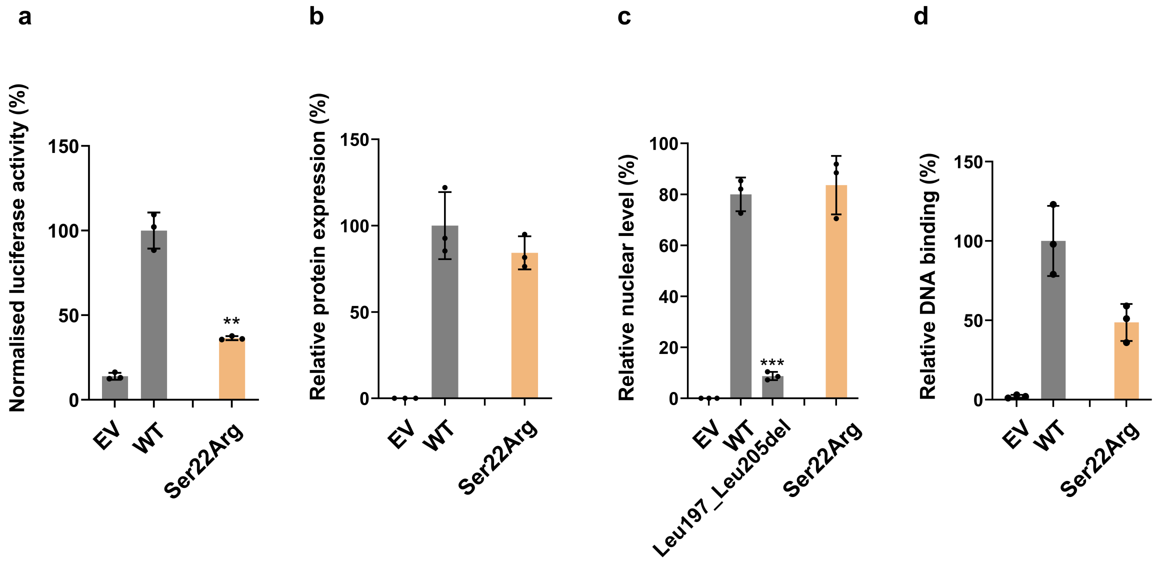


**ESM Fig. 6. Functional investigation of the p.Ser22Arg HNF1A variant identified in a positive autoantibody carrier. (a)** Assessment of transcriptional activity of HNF1A protein variants using a luciferase reporter assay. HeLa cells were transiently transfected with wild-type or variant *HNF1A* plasmids together with the reporter plasmids encoding *Firefly* (pGL3-RA) and *Renilla* (pRL-SV40) luciferase. **(b)** Relative protein expression. HeLa cell lysates collected for the transactivation assay were analysed by SDS-PAGE and immunoblotting using HNF1A specific antibodies. Results of protein levels, normalised to α-tubulin (loading control), are presented relative to wild-type levels (set as 100%). **(c)** Nuclear localisation of HNF1A variants. Nuclear fractions of transiently transfected HeLa cells (wild-type or variant *HNF1A* plasmids) assessed by SDS PAGE immunoblotting. p.Leu197_Leu205del was used as a negative control, and topoisomerase IIα and α-tubulin were used as nuclear and cytosolic markers, respectively. **(d)** DNA binding of HNF1A variants in electrophoretic mobility shift assay. Equal amounts of HNF1A variants in nuclear fractions were incubated with a Cy5 labelled oligo, corresponding to the HNF1A binding, and bound complexes quantified by densiometric analysis. Measurements are given relative to wild-type activity (set as 100%) unless otherwise specified. EV: empty vector. WT: wild-type. Each bar represents the mean of nine readings ± SD; three parallel readings conducted on three experimental days. The symbol ** indicates $p < 0.01$, and *** indicates $p < 0.001$.

# References

1. Danne T, Nimri R, Battelino T, Bergenstal RM, Close KL, DeVries JH, Garg S, Heinemann L, Hirsch I, Amiel SA, Beck R, Bosi E, Buckingham B, Cobelli C, Dassau E, Doyle FJ, 3rd, Heller S, Hovorka R, Jia W, Jones T, Kordonouri O, Kovatchev B, Kowalski A, Laffel L, Maahs D, Murphy HR, Nørgaard K, Parkin CG, Renard E, Saboo B, Scharf M, Tamborlane WV, Weinzimer SA, Phillip M. International Consensus on Use of Continuous Glucose Monitoring. Diabetes Care 2017;40:1631-1640
